# Supplementary material for: The interactome of intact mitochondria by cross-linking mass spectrometry provides evidence for coexisting respiratory supercomplexes
Source: Mol Cell Proteomics. 2017 Dec 8;17(2):216–32. doi: 10.1074/mcp.RA117.000470 (PMC5795388; doi:10.1074/mcp.RA117.000470)
Supplement: Supplemental Data [file supp_RA117.000470_133922_0_supp_23978_fzffwf.zip › spectra_annotation/mito_spectra_annotation/411-1-1-1-14-1.pdf]

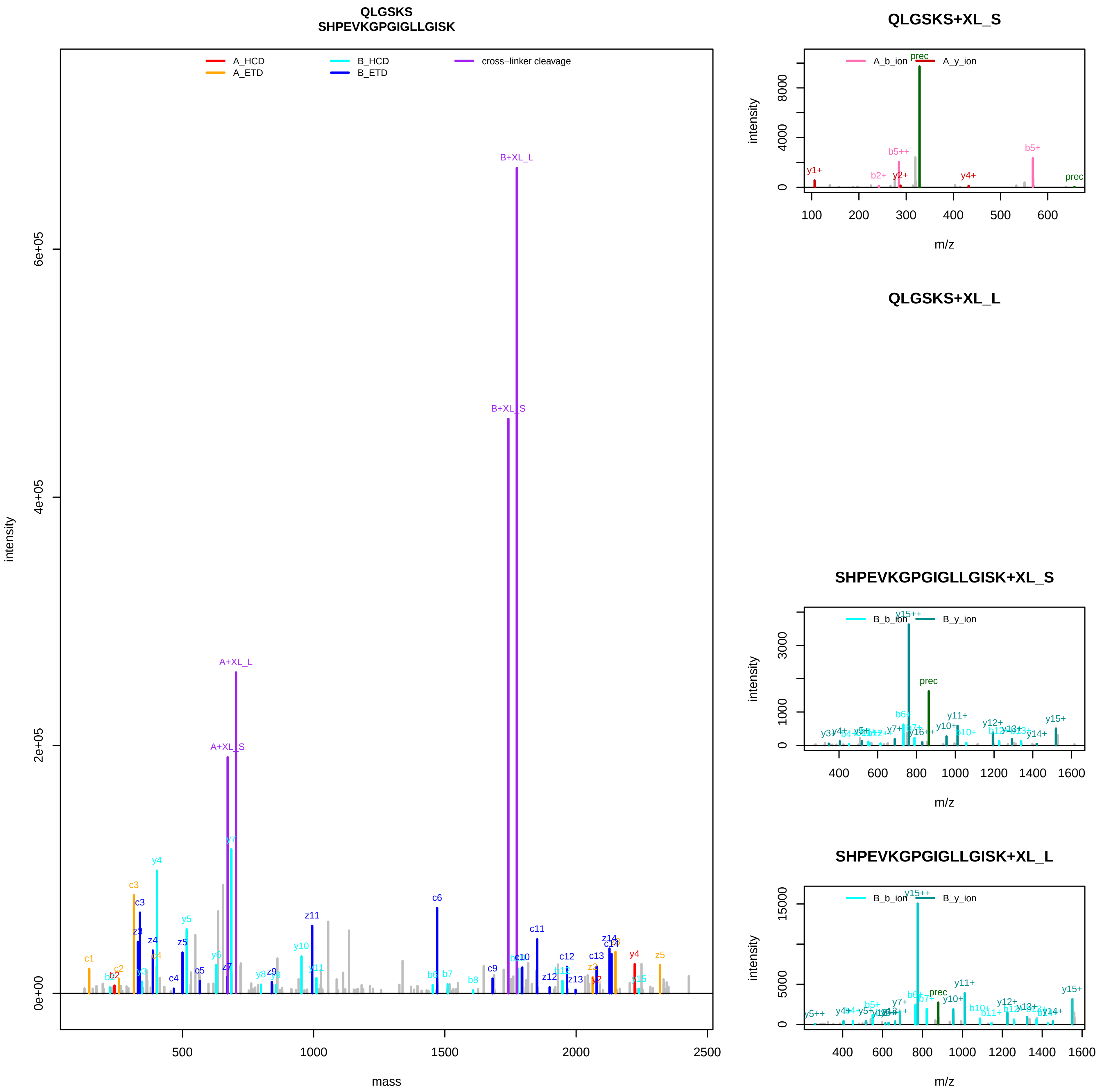
QLGSKS
SHPEVKGPGIGLLGISK
A_HCD
A_ETD
B_HCD
B_ETD
cross−linker cleavage
B+XL_L
B+XL_S
A+XL_L
A+XL_S
intensity
mass
0e+00
2e+05
4e+05
6e+05
500
1000
1500
2000
2500
c1
c2
b2
c3
z3
y3
c4
y4
z4
c4
z5
y5
c5
y6
z7
y7
y8
z9
y9
y10
z11
y11
c6
b6
b7
b8
c9
c10
c11
z12
c12
z13
c13
z14
c14
z2
y2
y4
y15
z5
QLGSKS+XL_S
A_b_ion
A_y_ion
prec
b5++
b2+
y2+
y1+
y4+
b5+
m/z
QLGSKS+XL_L
SHPEVKGPGIGLLGISK+XL_S
B_b_ion
B_y_ion
y15++
y11+
y10+
y12+
y13+
y14+
y15+
y16++
SHPEVKGPGIGLLGISK+XL_L
